# Supplementary material for: Maximizing Equity in Acute Coronary Syndrome Screening across Sociodemographic Characteristics of Patients
Source: Diagnostics (Basel). 2023 Jun 14;13(12):2053. doi: 10.3390/diagnostics13122053 (PMC10297640; doi:10.3390/diagnostics13122053)
Supplement: Supplementary file 1 [file diagnostics-13-02053-s001.zip › diagnostics-2374485-supplementary.pdf]

diagnostics

Supplementary Materials: Maximizing Equity in Acute Coronary Syndrome Screening Across the Sociodemographic Characteristics of Patients

Gabrielle Bunney, Sean M. Bloos, Anna Graber, Melissa Pasao, Rana Kabeer, David Kim, Kate Miller and Maame Yaa A. B. Yiadom

SUPPLEMENT A

Approach to the predictive model screening for acute coronary syndrome to identify those who need an electrocardiogram to identify STEMI

Predictive Modeling Method

The logistic regression model was of the form:

$$\text{Logit (ACS)} = \beta_0 + \beta_1 \text{ (chest pain)} + \beta_2 \text{ (other ACS symptom)} + \beta_3 \text{ (age)} + \beta_4 \text{ (female)} + \varepsilon$$

where ACS = whether diagnosed with ACS at the time of discharge (1 = yes, 0 = no),
chest pain = whether reported chest pain on arrival (0 = yes, 1 = no), with yes as the reference category,
other ACS symptom = whether reported another ACS symptom on arrival (0 = yes, 1 = no)), with yes as the reference category,
age = age in years (continuous),
female = sex (1 = female, 0 = male), and
ε = error term.

Since this model was fit to simulate a potential screening approach, we did not add or remove terms to tune the model fit.

This model yielded a predicted probability of ACS for each patient, ranging from 0% to 100%. In observed practice, 22% of patients received an early ECG, so that proportion was clearly feasible from an operational and cost perspective. To match that level of feasibility, we ordered all patients according to the model's predicted probability of ACS and selected the top 22% from this list as screen positives. Thus, our threshold for a positive screen was the 78th percentile of ACS risk. The final model equation was:

$$\text{Logit (ACS)} = -5.3668 + (-3.0571 * \text{no chest pain}) + (-0.3169 * \text{no other ACS symptom}) + (0.045 * \text{age}) + (-0.6438 * \text{female})$$

Measuring Screening Outcomes

For each subgroup, we constructed a standard 2x2 table of this form:

		True ACS status		
		Positive	Negative	Total
Screening status	Positive	A	B	A+B
	Negative	C	D	C+D
	Total	A+C	B+D	A+B+C+D

We calculated each outcome as follows:

Sensitivity = A / A+C

Specificity = D / B+D

Number of ECGs required = A+B

Number of missed ACS cases = C

Percent of missed ACS cases = C / (A+C)

Number of missed STEMI cases (drawn from the 2x2 table using true STEMI status in place of ACS) = C

Percent of missed STEMI (drawn from the 2x2 table using true STEMI status in place of ACS) cases = C / (A+C)

SUPPLEMENT B

Confidence intervals and point estimates associated with Figure 2: Sensitivity and specificity of ACS screening outcomes for each approach across sex, race, ethnicity, language, and age demographic subgroups.

Supplement B Table S1. Sensitivity of ACS screening outcomes for each approach across sex, race, ethnicity, language and age demographic subgroups.

**Sensitivity (95% CI)**		Screen 1: Observed Practice	Screen 2: Clinical protocol	Screen 3: Predictive model	Screen 4: Augmented human performance
Total		73.2% (70.8% - 75.5%)	55.5% (52.9% - 58.2%)	81.9% (79.8% - 83.9%)	92.4% (90.9% - 93.7%)
Sex	Female	69.3% (65.2% - 73.1%)	64.6% (60.4% - 68.5%)	75.8% (72.0% - 79.3%)	89.9% (87.1% - 92.3%)
	Male	75.8% (72.8% - 78.7%)	49.6% (46.2% - 53.1%)	85.9% (83.4% - 88.2%)	94.1% (92.3% - 95.6%)
Race	White	73.5% (70.0% - 76.7%)	61.4% (57.6% - 65.0%)	83.4% (80.5% - 86.1%)	93.7% (91.6% - 95.4%)
	Other race	73.7% (68.3% - 78.7%)	47.8% (41.9% - 53.7%)	82.6% (77.8% - 86.8%)	90.4% (86.5% - 93.6%)
	Asian	73.2% (67.3% - 78.6%)	60.8% (54.4% - 66.9%)	79.6% (74.1% - 84.4%)	90.4% (86.1% - 93.8%)
	Black	71.0% (60.6% - 79.9%)	36.6% (26.8% - 47.2%)	74.2% (64.1% - 82.7%)	92.5% (85.1% - 96.9%)
	NAAH/PI	72.3% (57.4% - 84.4%)	34.0% (20.9% - 49.3%)	76.6% (62.0% - 87.7%)	93.6% (82.5% - 98.7%)
Hispanic/ Latino	Yes	69.0% (62.1% - 75.3%)	40.5% (33.6% - 47.7%)	79.5% (73.2% - 84.9%)	87.5% (82.1% - 91.7%)
	No	74.0% (71.4% - 76.5%)	58.1% (55.2% - 61.0%)	82.1% (79.8% - 84.2%)	93.1% (91.5% - 94.5%)
Language	English	74.4% (71.8% - 76.9%)	53.8% (50.9% - 56.8%)	81.6% (79.2% - 83.8%)	92.9% (91.2% - 94.3%)
	Spanish	67.7% (58.8% - 75.9%)	46.8% (37.8% - 55.9%)	79.8% (71.7% - 86.5%)	88.7% (81.8% - 93.7%)
	Other	68.6% (60.2% - 76.1%)	77.1% (69.3% - 83.8%)	86.4% (79.6% - 91.6%)	92.1% (86.4% - 96.0%)
Age	<50	77.7% (70.4% - 84.0%)	0.0% (0.0% - 0.0%)	76.4% (69.0% - 82.8%)	86.6% (80.3% - 91.5%)
	50-64	77.1% (72.8% - 81.1%)	0.0% (0.0% - 0.0%)	71.8% (67.2% - 76.1%)	88.8% (85.4% - 91.7%)

	65-79	73.3% (69.1% - 77.1%)	91.9% (89.1% - 94.2%)	80.6% (76.8% - 84.0%)	92.7% (90.1% - 94.8%)
	80+	66.3% (60.9% - 71.3%)	96.1% (93.5% - 97.9%)	98.8% (97.0% - 99.7%)	99.1% (97.4% - 99.8%)

NAAH/PI = Native American, Alaskan, or Hawaiian/Pacific Islander.

Supplement B Table S2 Specificity of ACS screening outcomes for each approach across sex, race, ethnicity, language, and age demographic subgroups.

**Specificity (95% CI)**		Screen 1: Observed Practice	Screen 2: Clinical protocol	Screen 3: Predictive model	Screen 4: Augmented human performance
Total		78.3% (78.2% - 78.5%)	83.4% (83.3% - 83.6%)	78.1% (77.9% - 78.2%)	67.3% (67.2% - 67.5%)
Sex	Female	79.2% (79.0% - 79.4%)	83.0% (82.8% - 83.2%)	83.8% (83.6% - 84.0%)	71.9% (71.7% - 72.1%)
	Male	77.3% (77.1% - 77.6%)	83.9% (83.7% - 84.1%)	71.0% (70.7% - 71.2%)	61.7% (61.4% - 61.9%)
Race	White	77.0% (76.7% - 77.2%)	77.9% (77.7% - 78.2%)	72.8% (72.5% - 73.0%)	62.2% (62.0% - 62.5%)
	Other race	81.5% (81.2% - 81.7%)	91.0% (90.8% - 91.2%)	85.3% (85.0% - 85.5%)	75.2% (74.9% - 75.5%)
	Asian	76.3% (75.9% - 76.7%)	79.5% (79.1% - 79.9%)	75.3% (74.8% - 75.7%)	63.5% (63.0% - 64.0%)
	Black	78.1% (77.6% - 78.6%)	89.4% (89.0% - 89.8%)	82.7% (82.2% - 83.2%)	71.2% (70.6% - 71.7%)
	NAAH/PI	75.6% (74.6% - 76.5%)	89.8% (89.2% - 90.5%)	83.9% (83.1% - 84.7%)	69.1% (68.1% - 70.1%)
Hispanic/ Latino	Yes	82.5% (82.2% - 82.8%)	92.7% (92.5% - 92.9%)	87.0% (86.7% - 87.2%)	77.2% (76.8% - 77.5%)
	No	76.9% (76.7% - 77.1%)	80.3% (80.1% - 80.5%)	75.1% (74.9% - 75.3%)	64.0% (63.8% - 64.2%)
Language	English	78.6% (78.4% - 78.7%)	84.3% (84.1% - 84.4%)	78.7% (78.5% - 78.8%)	68.0% (67.8% - 68.1%)
	Spanish	80.9% (80.5% - 81.4%)	88.5% (88.1% - 88.8%)	83.7% (83.3% - 84.1%)	73.4% (72.9% - 73.9%)
	Other	69.8% (69.1% - 70.5%)	60.8% (60.1% - 61.6%)	58.5% (57.8% - 59.3%)	46.4% (45.6% - 47.1%)
Age	<50	85.1% (85.0% - 85.3%)	100.0% (100.0% - 100.0%)	92.9% (92.7% - 93.0%)	83.7% (83.5% - 83.9%)
	50-64	75.5% (75.1% - 75.8%)	100.0% (100.0% - 100.0%)	90.5% (90.3% - 90.7%)	73.5% (73.2% - 73.9%)
	65-79	70.1% (69.7% - 70.5%)	42.8% (42.4% - 43.3%)	59.1% (58.7% - 59.5%)	46.4% (46.0% - 46.9%)
	80+	64.9% (64.3% - 65.4%)	35.2% (34.6% - 35.7%)	9.3% (8.9% - 9.6%)	7.9% (7.5% - 8.2%)

NAAH/PI = Native American, Alaskan, or Hawaiian/Pacific Islander.
